# Epidemiologic patterns in HIV infection in Mureş county

**DOI:** 10.1186/1471-2334-14-S4-P6

**Published:** 2014-05-29

**Authors:** Lucia Carmen Chiriac, Erzsébet Iringó Zaharia-Kézdi, Brînduşa Ţilea, Anca Georgescu, Cristina Gîrbovan, Andrea Incze, Nina-Ioana Șincu, Ioana Palcu

**Affiliations:** 1 Department of Infectious Diseases, University of Medicine and Pharmacy, Tîrgu Mureş, Romania; 2 Infectious Diseases Clinic I, Tîrgu Mureş County Clinic Hospital, Tîrgu Mureş, Romania

## 

Despite the important scientific progress regarding HIV pandemics registered during the last decade, more and more cases of HIV infection are diagnosed every year, as HIV testing practices vary across Europe. Purpose: to identify epidemiologic trends in newly diagnosed patients with HIV infection in the center of Romania.

We performed a retrospective, cross-sectional study, over a 5-year period (January 2009 – December 2013), upon all new cases of HIV infection, diagnosed in Mureş county. We analyzed demographic data, transmission patterns, level of CD4 T-lymphocytes and patients’ outcome. Over a 5 years-period, we have diagnosed 58 new cases of HIV infection, 63.79% male / 36.21% female. Average age was 27 years, median – 23 years, with extremes between 2 and 55 years. Most cases came from urban areas (65.38%). Transmission patterns included risk groups: men who have sex with men (MSM) – 4 patients, unknown multiple partners – 3 patients, HIV-infected sexual partner – 15 patients, screening at delivery – 4 patients, 1 case of mother-to-child transmission. The dominant pattern was heterosexual transmission – 27 patients. CD4 T-cells count registered an average level of 369 cells/μL, median 335 cells/μL, ranging from 2 to 1304 cells/μL. 23 (39.65%) patients were late presenters. We registered 3 deaths (5.17%).

While previous epidemiologic data from our region suggested an unknown route of transmission in children born around the 1990’s, during the last years, the dominant epidemiologic pattern in HIV infection in Mureş county was heterosexual transmission. An important category of newly diagnosed patients was represented by late presenters, with potential unfavorable evolution. We registered a concerning trend of sexual transmission of HIV infection in serodiscordant couples, despite medical education.

